# Effect of fish oil and vitamin E on sperm lipid peroxidation in dogs

**DOI:** 10.1017/jns.2017.29

**Published:** 2017-09-11

**Authors:** Analía Lorena Risso, Francisco J. Pellegrino, Yanina Corrada, Mónica Marmunti, Mariana Gavazza, Alejandro Palacios, Alejandro E. Relling

**Affiliations:** 1 School of Veterinary Sciences, National University of La Plata, Buenos Aires, Argentina; 2 Institute of Veterinary Genetic Institute (IGEVET), UNLP/CONICET, La Plata, Buenos Aires, Argentina; 3 National Council of Research and Technology (CONICET), La Plata, Buenos Aires, Argentina; 4 Department of Animal Sciences, The Ohio State University, Wooster, OH 44691, USA

**Keywords:** Dog nutrition, Fish oil, Vitamin E, Lipid peroxidation, BW, body weight, CG, control group, cpm, counts per min, FEG, fish oil and vitamin E group, FG, fish oil group, FO, fish oil, LP, lipid peroxidation, VE, vitamin E.

## Abstract

The objective was to evaluate the effects of dietary fish oil (FO) and vitamin E (VE) supplementation on sperm sensitivity to lipid peroxidation (LP) in dogs. Using an incomplete replicate 3 × 3 Latin square design, five dogs were allocated into three groups. One of the squares was incomplete and had two dogs that were used with three treatments. The dogs were assigned to three different treatments, fed a control diet of balanced commercial food (control group; CG), control diet supplemented with 54 mg FO/kg body weight^0·75^ per d (FO group; FG) and FO plus 400 mg VE per d (FO and VE group; FEG) for 60 d. Semen samples were collected on days 0 and 60 and divided into two halves, peroxidised and control, with or without ascorbate–Fe^2+^, respectively. LP was measured in both halves by chemiluminescence as counts per min/mg protein. Fatty acid profile was determined by GC. Data were analysed using the mixed procedure (SAS). On day 0, LP increased in all groups in the peroxidised samples (*P* < 0·05). However, on day 60 LP decreased in peroxidised samples of both the FG and FEG (*P* < 0·05), but there were no differences between the FG and FEG (*P* > 0·1). Additionally, on day 60 total *n*-3 was higher in the FG and FEG compared with the CG (*P* < 0·05). Supplementation with FO alone or together with VE decreased LP in peroxidised samples. These results could indicate a protective effect of *n*-3 on sperm. More studies are needed to understand the mechanism whereby FO and/or FO plus VE decrease LP in dogs’ sperm.

Fish oils (FO) contain *n*-3 PUFA and are a major source of EPA (20 : 5*n*-3) and DHA (22 : 6*n*-3). In dogs, FO supplementation has been used for improving semen quality^(^^1^^)^. In addition, an increase of *n*-3 PUFA in dogs’ sperm after FO supplementation has been reported^(^^1^^)^.

Highly unsaturated fatty acids such as EPA, DHA and arachidonic acid (AA, 20 : 4*n*-6) have a high risk of oxidation^(^^2^^)^. Mammalian sperm are sensitive to lipid peroxidation (LP). They not only contain a high proportion of PUFA within the plasma membrane, but also contain an inadequate quantity of antioxidative enzymes^(^^3^^)^. LP damage to the sperm cell membrane results from the generation of reactive oxygen species such as the superoxide anion, hydroxyl radical and hydrogen peroxide. LP may induce loss of cell membrane integrity, thus increasing cell membrane permeability, enzyme inactivation, DNA structural damage and cell death^(^^4^^)^. Some LP products are light-emitting species and their spontaneous chemiluminescence can be used as an internal marker of oxidative stress^(^^5^^,^^6^^)^.

FO supplementation leads to the incorporation of EPA and DHA into the cell membrane^(^^2^^)^. It has been proposed that adequate antioxidant concentrations are necessary to match increased dietary intakes of *n*-3 PUFA^(^^7^^)^. Effects of LP can be avoided by supplementing diets enriched in *n*-3 PUFA with antioxidants such as vitamin E (VE). However, more recently, studies conducted in rats showed that dietary *n*-3 PUFA supplementation had antioxidative effects^(^^8^^)^.

To our knowledge, no studies have specifically evaluated LP in sperm of dogs supplemented with FO or FO plus VE. Therefore, the objective of this study was to evaluate the effects of dietary FO and VE supplementation on sperm sensitivity to LP in dogs. We hypothesised that FO supplementation would produce higher LP than FO plus VE in dogs’ sperm.

## Experimental methods

### Animals and treatments

The study was approved by the Institutional Animal Care and Use Committee (number 34-1-13) of the School of Veterinary Sciences, National University of La Plata, Buenos Aires, Argentina. The dogs in the study were selected according to age, body condition and a complete medical record (history, clinical visit and clinical examination). Additionally, as complementary methods, routine blood tests, chemical tests and semen evaluations were performed.

At 1 month before the study the dogs were adapted to a control diet of balanced commercial food. Daily rations of food were controlled and the amount for each dog was calculated using the formula for maintenance energetic requirements (MER): MER = (130 × kg metabolic body weight (BW^0·75^))^(^^9^^)^. Water was given *ad libitum*. The nutrient composition of the balanced commercial food in DM (%) was 30·04 protein, 15·02 fat, 1·72 fibre, 7·83 ash, 1·50 Ca, 1·07 P, 0·048 mineral and vitamin mix (12·39 VE, 0·20 vitamin K, 0·82 vitamin B_1_, 0·82 vitamin B_2_, 0·82 vitamin B_6_, 0·0004 vitamin B_12_, 0·12 folic acid, 0·10 nicotinic acid, 2·06 calcium pantothenate, 0·02 biotin, 82·1 choline, 0·01 Cu, 0·01 Fe, 0·02 Zn, 0·003 I, 0·01 Mn and 0·0002 Se).

Using an incomplete replicate 3 × 3 Latin square design, five 2- to 5-year-old male mixed-breed dogs with a body condition score of 3 in a 5 scale^(^^10^^)^ and 20–30 kg of BW were randomly allocated into three groups. One of the squares was incomplete and had two dogs that were used with three treatments. The duration of each period was 60 d. All the dogs started the study at the same time. In the first group, the dogs were fed a control diet of balanced commercial food without any supplement (control group; CG). In the second group, the dogs were fed the same diet as the CG supplemented with a capsule containing 54 mg FO/kg metabolic BW per d (FO group; FG). In the third group, the dogs received the same diet as the FG plus 400 mg VE per d (FO and VE group; FEG). Data of all three groups were collected at the same time (days 0 and 60).

During the study the dogs were kept at the owners’ homes. Owners agreed to feed the provided diet and supplement it with FO capsules and/or VE as required.

Finally, we included a 60 d wash-out period between treatments to avoid any carry-over effects, during which all dogs received the control diet.

### Preparation of canine semen samples

Previous to the study, the dogs were trained for semen collection, which was performed by manual stimulation twice a week. A 1 ml aliquot of semen from each sample was centrifuged at 800 ***g*** for 10 min. Sperm pellets were separated and washed by suspending in PBS and centrifuging (three times). After the last centrifugation, 1 ml of deionised water was added to sperm pellets^(^^11^^)^; they were snap-frozen and stored at −83°C. Sperm samples were used within 1 week of preparation, after one cycle of freezing and thawing. All operations were performed at 4°C.

### Non-enzymic lipid peroxidation of sperm

Sperm samples were divided in two halves (peroxidised and control), with or without ascorbate–Fe^2+^, respectively. Chemiluminescence and LP were initiated by adding ascorbate–Fe^2+^ to sperm samples^(^^12^^)^. Sperm samples (1 mg of protein) were incubated at 37°C with 0·01 m-phosphate buffer (pH 7·4) and 0·4 mm-ascorbate. The resulting solution was 1 ml. Fe was added into the phosphate buffer to provide the necessary ferrous or ferric iron for LP (final concentration in the incubation mixture was 2·15 µm)^(^^13^^)^. Sperm samples without ascorbate–Fe^2+^ (control) were carried out simultaneously. Chemiluminescence was measured as counts per min (cpm) in a liquid scintillation analyser (Packard 1900 TR)^(^^6^^)^. Membrane light emission was determined over a 120-min period and recorded as cpm every 10 min. The sum of the total chemiluminescence was used to calculate cpm/mg protein.

### Fatty acid composition of food, fish oil and sperm

Food, FO and sperm were analysed to evaluate the fatty acid composition. Sperm samples of each group were collected for lipid analysis on days 0 and 60 and stored at −80°C until analysis. Samples for lipid extraction were analysed following the method described by Folch *et al*.^(^^14^^)^. Once lipids were obtained, they were saponified with potassium hydroxide dissolved in ethanol for 45 min at 80°C, and then acidified with a 0·5 ml hydrochloric acid concentrate. The acids were esterified with boron trifluoride at 64°C for 1·5 h. Fatty acid composition was determined by GLC with a 30 mm capillary column (Omega Wax 250; Supelco). Temperature was programmed for a linear increase of 3°C per min from 175 to 230°C (Table 1).

**Table 1.** Fatty acid composition (%) of balanced commercial food and fish oil

Fatty acid	Balanced commercial food	Fish oil
Linoleic acid (18 : 2*n*-6)	38·1	2·0
α-Linolenic acid (18 : 3*n*-3)	4·0	1·1
Arachidonic acid (20 : 4*n*-6)	1·4	1·0
EPA (20 : 5*n*-3)	0·1	7·6
DHA (22 : 6*n*-3)	0·2	14·9
∑*n*-3	4·9	25·6
∑*n*-6	41·3	3·0

### Statistical analysis

The experimental design was an incomplete replicate 3 × 3 Latin square. Each individual dog was considered an experimental unit. One of the squares was incomplete and had two dogs that were used with three treatments. Data were analysed with the PROC MIXED of SAS (version 9.0; SAS Institute Inc.), with repeated measurements. The repeated measurement was comparing the same semen sample LP with and without ascorbate–Fe^2+^. The model included the random effect of dogs, the period of Latin square and the Latin square replicate, and the fixed effect of treatment (CG *v*. FG *v*. FEG), peroxidation and the interaction. Because none of the interactions was significant (*P* > 0·1), the interaction was removed from the model. Mean separation of the treatment was done using an orthogonal contrast comparing CG *v*. FG and FEG, and FG *v.* FEG. For day 60 values, the values obtained on day 0 were used as co-variables. Data are represented as least square means with their standard errors. The α level of significance was set at *P* < 0·05.

## Results

Comparison of control with ascorbate–Fe^2+^-treated samples showed a significant increase in light emission (chemiluminescence). On day 0, LP increased significantly in peroxidised (with ascorbate–Fe^2+^) samples in all groups (*P* < 0·05). This increase was the same in the three groups (*P* > 0·1). LP values were 429·00 (sem 60) and 990·50 (sem 87) cpm in the control and peroxidised sperm samples, respectively. However, on day 60 there was a decreased LP in peroxidised samples of both the FG and FEG (*P* < 0·05) comparing with the CG, but there were no differences between the FG and FEG (*P* > 0·1) (Table 2).

**Table 2.** Lipid peroxidation (counts per min/mg protein) in control (without ascorbate–Fe^2+^) and peroxidised (with ascorbate–Fe^2+^) semen samples and total *n*-3 and *n*-6 fatty acid content (%) in sperm samples of five dogs in the control group (CG, *n* 5), fish oil group (FG, *n* 5) and fish oil plus vitamin E group (FEG, *n* 5) on day 60 (Least square means (LSM) with their standard errors)

	Control		Peroxidised		Total *n*-3		Total *n*-6	
Treatment	LSM	sem	LSM	sem	LSM	sem	LSM	sem
CG	379·20^a^	41	900·50^b^	87	5·12^b^	0·29	34·30^a^	1·40
FG	436·00^a^	41	538·80^a^	41	7·73^a^	0·29	33·50^a^	1·40
FEG	301·04^a^	45	532·40^a^	41	8·05^a^	0·29	33·80^a^	1·40

^a,b^ Mean values within a column with unlike superscript letters were significantly different (*P* < 0·05).

In addition, on day 0 there were no differences in total *n*-3 and *n*-6 fatty acid content in sperm samples of the three groups (*P* > 0·1). The values (%) were as follows: CG: total *n*-3, 5·28 (sem 0·32) and total *n*-6, 35·20 (sem 1·20); FG: total *n*-3, 5·10 (sem 0·32) and total *n*-6, 34·26 (sem 1·20); FEG: total *n*-3, 5·40 (sem0·32) and total *n*-6, 34·90 (sem 1·20). Conversely, on day 60 there were significant differences in total *n*-3 in the FG and FEG. Percentage total *n*-3 was higher in the FG and FEG compared with the CG (*P* < 0·05) (Table 2).

## Discussion

In the present study, we assessed the effect of dietary supplementation with FO either alone or with VE on dog sperm LP. Additionally, we measured fatty acid profile in a balanced commercial food, in the FO supplement and in sperm samples. Contrary to our expectations, supplementation with FO alone or together with VE decreased sperm LP on day 60.

Lenox & Bauer^(^^2^^)^ stated that the inclusion of VE as an antioxidant may be needed in conjunction with FO supplementation in the diet to avoid the effect of LP. In the present study, there were no differences between the FG and FEG. In agreement with that reported by LeBlanc *et al*.^(^^15^^)^ who evaluated the production of hydroperoxides in plasma, dietary supplementation with FO with or without the addition of VE did not produce differences between groups. However, opposed to the findings of LeBlanc *et al*.^(^^15^^)^, our results showed a decrease in LP in both treated groups. Differences could be due to the dose administered, the duration of supplementation and/or the FO source used. The National Research Council^(^^16^^)^ mentions that diet VE concentrations necessary to protect against cell membrane LP depend on the concentration of fat in the diet, the proportion of PUFA, the concentration of Se, the degree of peroxidation of these fatty acids and the presence of other antioxidants. In our experiment, the percentage of fat, PUFA and Se in the balanced commercial food was in accordance with recommendations^(^^16^^)^. The peroxide index of FO was in the normal range (2·5 meq/kg). Therefore, addition of 400 mg VE to this balanced food supplemented with 54 mg FO/kg metabolic BW would be unnecessary and FO alone would account for these results. On the other hand, the increase in the proportion of *n*-3 in sperm samples of the FG and FEG may indicate a protective effect of *n*-3 which leads to less LP. In this regard, Jones *et al*.^(^^8^^)^ demonstrated that supplementation with *n*-3 PUFA reduced oxidative stress, as evidenced by a decrease in LP.

We concluded from this study that supplementation with FO alone or together with VE decrease LP in peroxidised samples. However, more studies are needed to understand the mechanism whereby FO and/or FO plus VE decreases LP in peroxidised samples in dogs’ sperm and to investigate the effects of FO with standardised diets on dogs and types of LP products produced.
